# It feels real: physiological responses to a stressful virtual reality environment and its impact on working memory

**DOI:** 10.1177/0269881119860156

**Published:** 2019-07-11

**Authors:** Marieke AG Martens, Angus Antley, Daniel Freeman, Mel Slater, Paul J Harrison, Elizabeth M Tunbridge

**Affiliations:** 1 Department of Psychiatry, University of Oxford, Warneford Hospital, Oxford, UK; 2 Oxford Health NHS Foundation Trust, Warneford Hospital, Oxford, UK; 3 Department of Clinical Psychology and Psychobiology, University of Barcelona, Barcelona, Spain

**Keywords:** Virtual reality, stress, cortisol

## Abstract

**Background:**

Virtual reality (VR) is increasingly used to study and treat psychiatric disorders. Its fidelity depends in part on the extent to which the VR environment provides a convincing simulation, for example whether a putatively stressful VR situation actually produces a stress response.

**Methods:**

We studied the stress response in 28 healthy men exposed either to a stressor VR elevator (which simulated travelling up the outside of a tall building and culminated in the participant being asked to step off the elevator platform), or to a control elevator. We measured psychological and physiological (salivary cortisol and alpha-amylase, blood pressure, pulse, skin conductance) stress indices. We also measured subsequent performance on the N-back task because acute stress has been reported to impact on working memory.

**Results:**

Compared to participants in the control elevator, those in the external elevator had increases in skin conductance, pulse and subjective stress and anxiety ratings, altered heart rate variability, and a delayed rise in cortisol. N-back performance was unaffected.

**Conclusions:**

A putatively stressful VR elevator produces a physiological as well as a psychological stress response, supporting its use in the investigation and treatment of stress-related disorders, and its potential value as an experimental laboratory stressor.

## Introduction

Virtual reality (VR) is increasingly being used to investigate and to treat psychological disorders, such as paranoia, social phobia, and acrophobia (Freeman et al., 2018; Freeman et al., 2019; Gujjar et al., 2019; Kampmann et al., 2016). The immersive nature of VR can simulate key aspects of the condition of interest, whilst allowing full control over the environment and the opportunity to undertake more intensive investigations than is possible in the real-world situation. However, for a VR analogue to be effective, the environment must be sufficiently convincing for the participant to experience a high degree of presence (Sanchez-Vives and Slater, 2005). Thus, if VR is to be used for disorders in which stress or anxiety are a key component, or indeed as a means to induce acute stress (e.g. for use in experimental medicine studies), it is important to ensure that the VR environment produces a bona fide stress response, and to understand its nature, magnitude and duration.

Several studies have measured components of the stress response to different VR environments (Diemer et al., 2014) but much remains unknown. As well as there being inconsistencies, no study has examined the full range of standard stress indices, nor have they compared them between-subjects in individuals randomised to a stressful vs. a control VR scenario (studies that have used control scenarios have examined the effect of control vs. stress scenarios using a within-subjects approach (e.g. Cleworth et al., 2012)). This information is critical if VR scenarios are to be of value as laboratory-based stressors in experimental medicine studies, since both within- and between-subjects designs are in routine use. For example, between-subjects designs are often necessary, for example, to minimise order or practice effects or to prevent dropout in long-term studies; however, they require that any stress intervention is robust enough to produce between-group differences.

The stress response is not a unitary construct; instead, it involves multiple interacting components. Central to the stress response is the activation of the autonomic nervous system (ANS) and the hypothalamic-pituitary-adrenal axis (HPA). The ANS, consisting of sympathetic and parasympathetic arms, provides the most immediate response to stressor exposure. Activation of the sympathetic arm represents the classic ‘fight or flight’ response: an increase in circulating catecholamines leads to a rapid (seconds) increase in heart rate (HR) and blood pressure (BP). Moreover, as a consequence of this autonomic activation, there is an increase in sweat gland activity (measured as skin conductance). However, through simultaneous parasympathetic activation, which generally acts to oppose sympathetic effects, this excitation is short-lived (de Kloet et al., 2005; Ulrich-Lai and Herman, 2009). Salivary alpha-amylase has been identified as a possible biomarker of ANS reactivity to stressors, particularly sympathetic activity (Ditzen et al., 2014; Petrakova et al., 2017). Activation of the HPA axis, which results in the secretion of cortisol, is slower, but longer-lasting (hours) depending on stressor intensity. The HPA axis and sympathetic system have largely complementary actions throughout the body, including energy mobilisation and maintenance of BP during the stress response.

Another measure associated with the stress response is heart rate variability (HRV). HRV is the fluctuation of the length of heart beat intervals and is sensitive to stressor-evoked changes in ANS activity. Relative sympathetic increases in HR cause the time between heart beats (the interval between successive R waves, the RR interval) to become shorter, and relative parasympathetic increases cause the interbeat interval to become longer. A variety of measures have been used use to operationalise HRV. For example, the root mean square of successive differences (RMSSD) in RR impacted by the parasympathetic arm of the ANS, decreases during stressor exposure, as increases in stress are associated with decreases in the RR interval. HRV can also be analysed in the frequency domain, which allows the autonomic balance to be quantified. The high-frequency (HF) domain is often used as a measure of parasympathetic activity, whilst low frequency (LF) reflects the activity of the sympathetic nervous system. Psychological stressors are associated with an increase in the LF/HF ratio (Camm et al., 1996). However, it should be noted that the relationship between HRV and ANS activity is complex and so this measure’s interpretation is not always straightforward (Billman, 2011; Billman, 2013; Shaffer and Ginsberg, 2017).

Here we compared a range of psychological and physiological stress indices between healthy individuals during a VR elevator ride, which is intended to be stressful, compared with a control VR elevator ride. We predicted that the stressful VR scenario would produce an acute sympathetic-mediated stress response (affecting pulse, BP, skin conductance, and salivary amylase) but, given the relatively brief duration of the stressor, that it would likely be insufficient to produce detectable elevations in cortisol. After the stressor, participants completed the N-back task of working memory, in light of prior data suggesting that acute stress can impact on memory performance (Henckens et al., 2009; Schoofs et al., 2008; Schwabe et al., 2012).

## Methods

### Participants

Ethical approval was granted by the Inter-Divisional Research Ethics Committee of the University of Oxford (MSD-IDREC-C2-2014-0022). Twenty-eight non-smoking, healthy men aged 18 to 45 were recruited by advertisement. All potential participants underwent initial telephone screening to establish inclusion and exclusion criteria. Mental health was assessed using the Structured Clinical Interview for DSM-IV. Exclusion criteria included a current or past history of psychiatric or neurological disorder (including height phobia); use of psychotropic medication or medication that may affect the stress response (e.g. corticosteroids, beta-blockers); a history of heart disease or hypertension; alcohol intake greater than 30 units/week; illicit drug use in the last 3 months, and an inadequate command of spoken English. Demographics for the 28 participants who passed screening (13 of whom were randomised to the control and 15 of whom were randomised to the stress condition) are given in Table 1.

**Table 1.** Demographic and personality measures.

	Control lift (mean ± SEM)	Stress lift (mean ± SEM)
Number	13	15
Age (years)	23.9 ± 1.0	25.9 ± 1.2
NART	113 ± 2.4	111 ± 1.9
Locus of control	75.4 ± 3.3	73.0 ± 3.5
Self-esteem	30.2 ± 1.8	30.2 ± 1.0
Computer literacy	5.1 ± 0.2	5.7 ± 0.3
Number of exposures to VR environment	1.4 ± 0.4	1.5 ± 0.5
Years in education	16.3 ± 0.9	18.4 ± 0.7

Values are mean ± standard error of the mean (SEM). NART = National Adult Reading Test; Locus of control: Rotter’s Locus of Control scale; Self-esteem: Rosenberg’s Self-Esteem Scale; Computer literacy + number of exposures to VR: Likert scale 1–7; Years in education from age 6.

**Figure 1.**
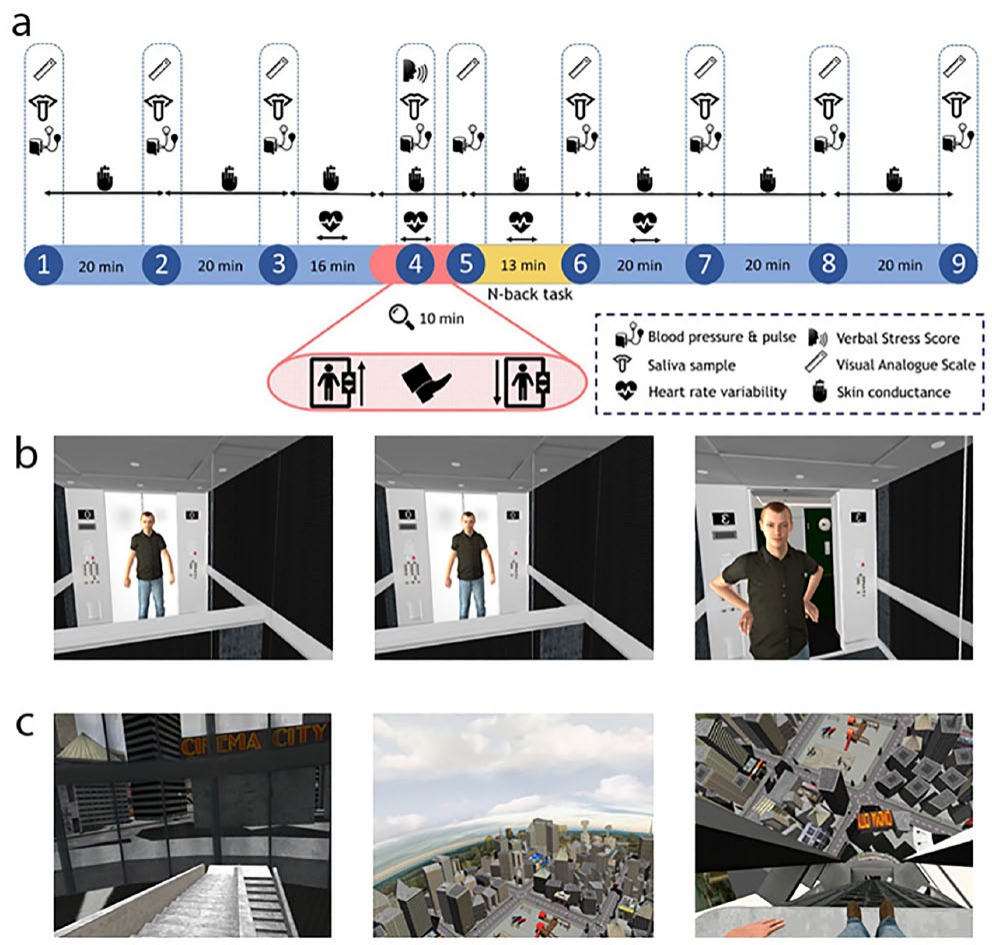
Summary of experimental manipulations. (a) Timeline of the experiment. Blue indicates pre- and post-testing periods; red indicates the time period during which participants were in the VR environment; yellow indicates the period in which participants completed the N-back task (outside of the VR environment). (b) Images from the control elevator. The avatar shown is that of the participant, viewed in a mirror (this reflection was only seen in the case of the control elevator). (c) Images from the stress elevator. For details, see text.

On the day of the experiment, participants were assigned to the control or stress VR scenario by simple randomisation (coin toss) by the experimenter. The procedure was conducted by a single researcher, in a single-blind manner. All instructions were delivered verbally. All participants knew they were taking part in a study that involved a VR scenario that could potentially stress them, but no further details were given until they were debriefed following data collection. Participants were instructed not to have any food or caffeinated drinks after 1 p.m., and attended the laboratory between 2 and 3 p. m., since cortisol levels are predicted to be most stable in the afternoon (Sin et al., 2017). Participants changed into a motion-tracker VR suit, and a BP cuff (Omron, used for both BP and pulse measures) was fitted. During a 1-h baseline period, they filled in several questionnaires: Rotter’s Locus of Control scale, the Rosenberg Self-Esteem Scale, and the National Adult Reading Test (NART), a measure of verbal IQ. Locus of control and self-esteem were measured since both have been reported to affect the magnitude of the response to an experimental stressor (Bollini et al., 2004, Pruessner et al., 1999). Participants were also asked how often they had been exposed to a VR environment, and were verbally instructed on the N-back. Participants then completed either the control or stressor version of the VR scenario, described below, before (following a brief verbal reminder of the instructions) completing the N-back task (described below). Following completion of the N-back task, participants remained in the laboratory for another hour, after which they were fully debriefed as to the nature and purpose of the experiment. A schematic of the experimental procedure is shown in Figure 1a.

### Virtual reality equipment

Participants were immersed in the VR world via a head-mounted display (nVisor SX111 HMD) and velcro Optitrack suit (with 37 tracking markers) linked to a computer and tracking system (12 Intersense SoniStrip ceiling and an InterSense IS-900 SimTracker) (Freeman et al., 2016).The head-mounted display combined a 102° horizontal field of view and 64° vertical field of view with a high resolution (1280 × 1024). A stereo image was presented using a screen for each eye, updated at 60Hz. The InterSense SoniStrip ceiling and InterSense IS-900 SimTracker system combined an inertial and time of flight audio sensor to specify the viewer’s position and orientation with 6 degrees of freedom. The resolution of the IS-900 was <0.75 mm, with an update rate of 180Hz, and latency of 4 msec. The computer running the application was custom built, and included a core i7 processor, and a NVIDIA GeForce GTX 780 ti graphics card with 3072MB of memory. This machine had 16GB RAM and an Asus Maximus VII Ranger motherboard. The tracking computer was a Dell T5500 workstation with a core i7 processor and 4GB RAM. We rendered audio using the Realtek audio controller provided by the motherboard.

### Virtual reality environments

There were two VR environments: an indoor closed elevator (control condition) and an outdoor open platform elevator (stress condition). For both scenarios, participants stood on a 3 cm thick wooden platform (85 × 40 inches) placed directly on the ground, whose edges corresponded to the edges of the VR elevator platform. Both elevator scenarios included two 15 s stops during the ascent and descent, and the sound of an elevator played. The ascent and descent took 100 s each; the total time of the VR paradigm was approximately 7.5 min.

The control scenario simulated an elevator ride from a ground-floor lobby up to the third floor; on arrival at the third floor, participants were instructed to step out of the lift and read the time on the clock in the hallway. The participant was alone, but was able to see their avatar’s live mirror image via a ‘mirror’ on the back wall of the elevator (Figure 1b). The stress scenario consisted of a VR journey on an open platform scaling the outside of a building, from the ground floor up to the top of a skyscraper, with an expanding view over the city (Figure 1c). Upon arriving at the top of the building, participants were instructed by the experimenter to step off the platform (i.e. as if falling) without hesitation. The edge of the platform corresponded to the edge of the virtual platform. Immediately after stepping out/off the elevator, BP and pulse rate (monitored for 30 s, during which time participants were stationary) were measured, and participants were asked to verbally rate how stressful they found the experience on a scale from 0 to 10. In both scenarios, the elevator then descended in a similar manner as it had ascended.

We recorded total movement (in metres) of each participant, to ensure that this was well-matched between the scenarios. For each of the 60 frames per second refreshes of the NVIS SX111 head-mounted display, the position was read from the IS-900 tracker and written to an output data file, which was used to calculate the total movement of each participant.

### Measurements of stress response

A range of psychological and physiological stress measures were taken throughout the experiment, as shown in Figure 1a. The first three time points (T1–3) were at 20-min intervals in the hour prior to VR exposure; T4 occurred during the VR, immediately after the participant stepped off/out of the elevator; T5 corresponded to the end of the VR; T6 was taken at the end of the N-back, and T7–T9 followed at 20-min intervals over the subsequent hour.

Subjective reports of alertness, drowsiness, stress, happiness, sadness, anxiety and nausea were assessed using visual analogue scales (VAS) at all time points except T4, since at this time point the participant was immersed in the VR scenario. A verbal rating (from 0–10) of stress was taken at T4 but was analysed separately.

Cortisol and alpha-amylase were determined in saliva collected using salivettes (SalivaBio Oral Swab Saliva Collection Method, Salimetrics, Carlsbad, CA, US). Within 4 h of collection, samples were centrifuged for 15 min at 3000 r/min, aliquoted, and frozen at −20°C. Cortisol and alpha-amylase concentrations were determined (in duplicate) using either a competitive immunoassay (cortisol; Salimetrics Assay ID: 1-3002) or a kinetic enzyme assay (alpha-amylase; Salimetrics Assay ID: 1-1902). Intra- and inter-assay variability was <7% for cortisol and <11% for alpha-amylase.

HR and skin conductance were continuously monitored throughout the experiment using Psychlab SC-EKG equipment at 500Hz. QRS detection used the Pan and Tompkins’s algorithm (Pan and Tompkins, 1985). From the heart rate data, HRV was calculated over four 5-min periods (before VR, during VR, during N-back and after N-back). For clarity, we present RMSSD (in the time domain, as an index of short-range HRV) and low-frequency–high-frequency ratio (LF-HF; in the frequency domain, which may reflect sympathetic modulations) as the main outcome measures (Camm et al., 1996). Skin conductance was measured using two 8-mm Ag/AgCl electrodes filled with isotonic electrolyte gel positioned on the index and middle finger of the non-dominant hand. Skin conductance was expressed as mean skin conductance level (µS) between the different data points.

### N-back

The N-back task imposes a parametric load on working memory. The version used here is relatively demanding (Farrell et al., 2012). It was delivered outside of the VR environment. Briefly, a number between 1 and 4 was randomly displayed on the computer screen (outside of the VR environment). For the 0-back condition, participants responded to the number showing on the screen by pressing the appropriate numerical button on the keyboard. For the 1-back, participants responded to the previous number on the screen, and so on for 2- and 3-back conditions. Each number was shown for 160 ms, with an interval of 1640 ms between numbers and 3000 ms between blocks. Six blocks of each condition were run. The primary performance measure was accuracy (correct responses); we also measured reaction time (RT) for correct trials. The task commenced within 2 min of completing the VR scenario, with a BP reading and VAS rating taken in between (T5).

### Statistical analyses

Analyses were carried out using SPSS for Windows (Version 22, IBM SPSS Statistics). Data were expressed and analysed as a percentage of baseline, except for subjective stress ratings (since some participants endorsed zero values at baseline precluding normalisation). ‘Baseline’ was defined as measurement T3, as cortisol levels were anticipated to decline over the course of the study period, as the result of circadian variation and acclimatisation to the laboratory setting.

### Main analyses

Since the autonomic stress response is brief, we pre-specified T4 (i.e. immediately after the acute stressor) as our primary time point of interest for BP, pulse, alpha-amylase and (verbally reported) subjective ratings of stress and anxiety. Similarly, skin conductance and HRV measures focussed on the period in the VR scenario as the main time interval of interest. The VAS ‘Stressed’ measure taken after the VR exposure (at T5), and the verbal ‘Stressed’ rating taken immediately after stepping off/out of the lift, were the primary psychological measures. In contrast, cortisol levels rise more slowly after stress, peaking after 20 min (Kudielka et al., 2009). Thus, we defined T6, which occurred approximately 20 min after the VR scenario, as our primary time point of interest for cortisol levels. Values at these predetermined time points were compared between conditions (control vs. stress) using *t*-tests or Mann–Whitney U tests where data were non-normally distributed (as assessed using Kolmogorov–Smirnov tests).

N-back data were analysed using repeated-measures analysis of variance (ANOVA), with level (0–3 back) as a within-subjects factor and group (stress or control) as the between-subjects factor.

### Exploratory analyses

We explored the effects of the VR environment on the psychological and physiological measures using repeated-measures ANOVA with time as the within-subjects factor and group as the between-subjects factor. Raw data (rather than percentage change from baseline) and all time points (including the three baseline measures) were used for these analyses; VAS, cortisol, skin conductance and HRV measures were log-transformed as they were non-normally distributed. Huynh-Feldt correction was applied where data failed Mauchly’s Test of Sphericity.

We conducted correlational analyses (Spearman’s rho, given the non-normal nature of many of the variables) to investigate the relationships between physiological and psychological variables, N-back performance, and personality characteristics. To limit the number of correlations examined, we focussed on the predetermined time points and measures included in the main analyses, with the addition of baseline (T3) VAS stress and anxiety scores, Rotter’s Locus of Control and Rosenberg’s self-esteem measures, and N-bank percent correct scores for each level.

## Results

Participant demographics are given in Table 1. All participants completed the study. There were no group differences in the distance that participants moved within the VR scenarios, either overall or at the top of the elevator (*t*s < 0.75; *p*s > 0.48). Due to technical difficulties, one participant (in the stress group) had missing ECG data, and three (one in the control and two in the stress group) had missing skin conductance data. One participant (in the stress group) did not complete the N-back, and another did not complete VAS ratings at T5.

### Main analyses

**Figure 2.**
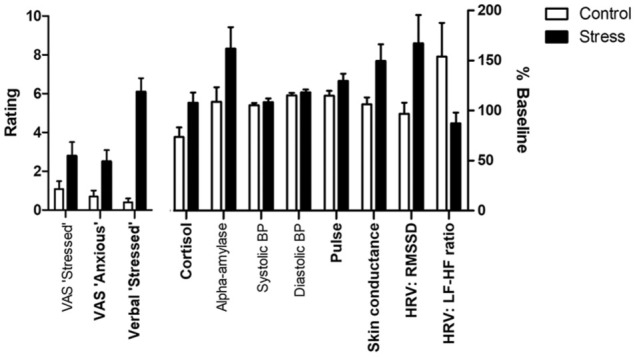
Summary of the main analyses, with key variables in those randomised to the control (open bars) or stress (closed bars) shown as means ± SEMs. Variables showing a group difference are highlighted with bold text (see Table 2 for full statistical details).

The results of the group comparisons for stress measures at the predetermined time points are shown in Figure 2. As shown in Table 2, compared to the control, participants exposed to the stress VR scenario reported greater stress and anxiety. There was an increase in pulse and skin conductance, and changes in HRV, but no change in BP. Unexpectedly, exposure to the stress VR scenario was sufficient to increase salivary cortisol, compared to the control. The increase in alpha-amylase achieved trend-level statistical significance.

**Table 2.** Effects of acute stress exposure in the VR scenario on physiological and psychological stress measures.

	Control (*n*; mean ± SEM)	Stress (*n*; mean ± SEM)	Statistic	Effect size*
VAS ‘Stressed’	*n* = 13; 1.09 ± 0.4	*n* = 14; 2.8 ± 0.7	*U* = 52; *p* = 0.058	0.365
VAS ‘Anxious’	*n* = 13; 0.7 ± 0.3	*n* = 14; 2.5 ± 0.6	*U* = 40; *p* = 0.013	0.477
Verbal ‘Stressed’ rating	*n* = 13; 0.4 ± 0.2	*n* = 15; 6.1 ± 0.7	*U* = 5; *p* = 0.000013	0.823
Cortisol (% baseline)	*n* = 13; 73.5 ± 9.6	*n* = 15; 107.9 ± 10.2	*t* = –2.4; *p* = 0.022	0.925
Alpha-amylase (% baseline)	*n* = 13; 108.7 ± 14.5	*n* = 14; 161.9 ± 21.3	*t* = –2.0; *p* = 0.056	0.770
Systolic BP (% baseline)	*n* = 13; 105.2 ± 2.4	*n* = 14; 108.6 ± 3.5	*U* = 75; *p* = 0.44	0.150
Diastolic BP (% baseline)	*n* = 13; 115.0 ± 2.6	*n* = 14; 118.3 ± 2.6	*U* = 74; *p* = 0.41	0.159
Pulse (% baseline)	*n* = 13; 114.8 ± 4.9	*n* = 14; 129.6 ± 7.1	*U* = 45; *p* = 0.026	0.430
Skin conductance (% baseline)	*n* = 12; 106.4 ± 6.6	*n* = 13; 149.6 ± 16.6	*U* = 35; *p* = 0.019	0.468
HRV: RMSSD (% baseline)	*n* = 13; 96.7 ± 11.2	*n* = 14;167.2 ± 28.2	*U* = 43; *p* = 0.019	0.448
HRV: LF-HF ratio (% baseline)	*n* = 13; 153.9 ± 33.8	*n* = 14;87.2 ± 10.9	*U* = 45; *p* = 0.025	0.430

SEM = standard error of the mean; BP = blood pressure; HRV = heart rate variability; LF-HF = low frequency–high frequency; RMSSD = root mean square of successive differences. *Effect size given as Z/√n for Mann–Whitney U; Cohen’s D for *t*-test.

In contrast to the physiological and psychological measures, there was no group difference in N-back performance (Table 3). Thus, whilst there were main effects of level for the percentage correct trials (*F*_3,75_ = 72.9; *p* = 6.2 × 10^-18^), there was no effect of level on RT (*F*_3,75_= 1.4; *p* = 0.27) and no main or interactive effects of group on either measure (*F*s < 3.3; *p*s > 0.57). There were no main or interactive effects for RT (*F*s < 1.4; *p*s > 0.26).

**Table 3.** N-back performance did not differ between groups.

	Control (Mean ± SEM)	Stress (Mean ± SEM)
0-back % correct	97.0 ± 0.6	97.5 ± 0.6
1-back % correct	84.4 ± 3.5	86.0 ± 3.3
2-back % correct	65.4 ± 5.0	70.2 ± 4.8
3-back % correct	52.4 ± 5.6	55.1 ± 5.4
0-back reaction time (msec)*	552.2 ± 35.8	539.6 ± 34.5
1-back reaction time (msec)*	491.9 ± 49.8	519.6 ± 49.8
2-back reaction time (msec)*	546.4 ± 57.7	590.7 ± 57.7
3-back reaction time (msec)*	553.2 ± 42.2	568.0 ± 40.7

* Reaction time calculated for correct trials only. SEM = standard error of the mean.

### Exploratory analyses

**Figure 3.**
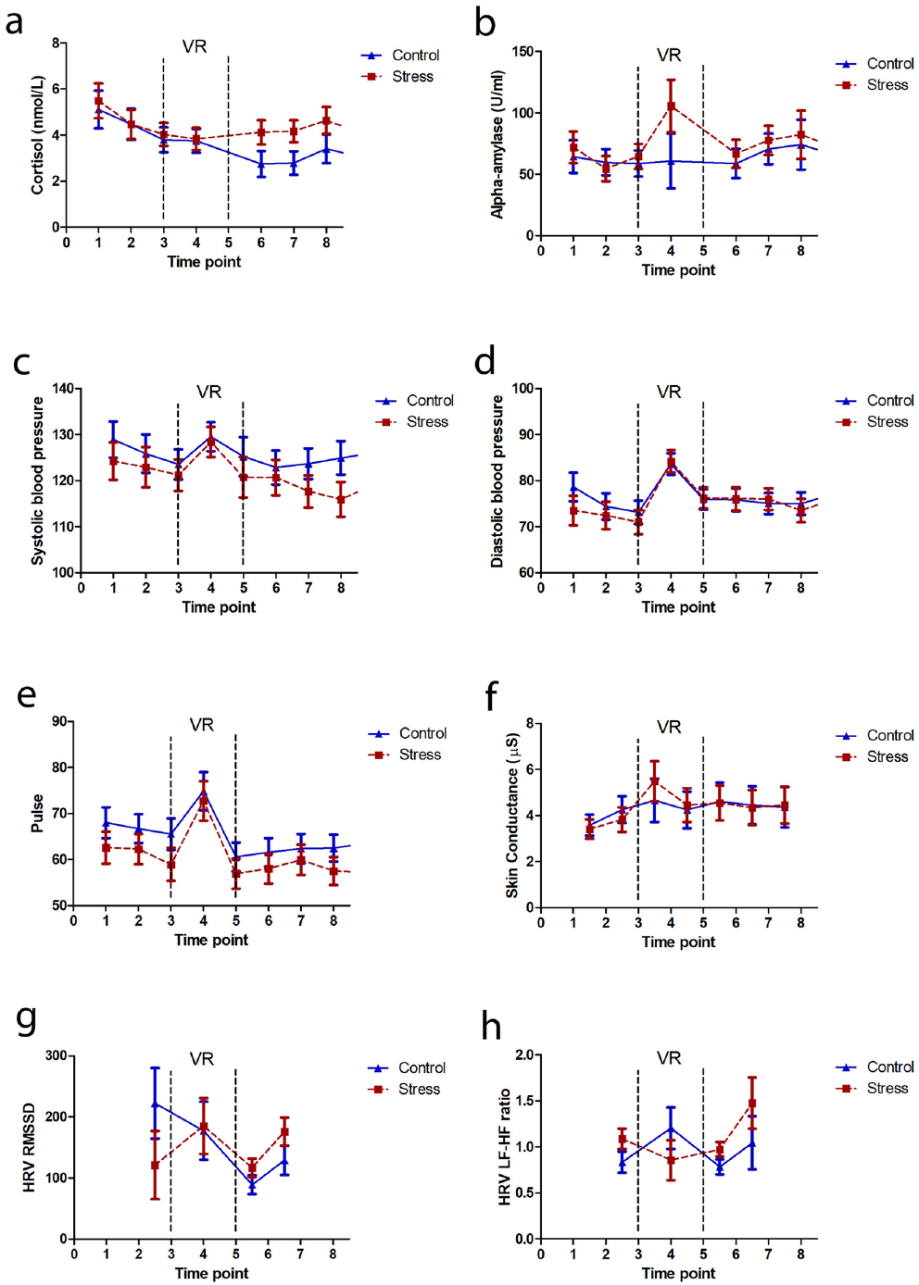
Variation in physiological stress indices across the experimental time course. Changes in (a) cortisol, (b) alpha-amylase, (c) systolic blood pressure, (d) diastolic blood pressure, (e) pulse rate, (f) skin conductance, (g) heart rate variability RMSSD and (h) LF-HF ratio measures over the course of the experiment in those randomised to the control (blue triangles and solid lines) and stress (red squares and dashed lines) elevator. Vertical dashed lines indicate the period that participants were immersed in the VR environment (i.e. from just after time point 3 to just before time point 5).

In the control group, cortisol levels showed the predicted diurnal decrease over the course of the experiment; in contrast, cortisol levels remained relatively constant in the stress group (Figure 3a). Thus, there was a main effect of time (*F*_7,182_ = 5.0; *p* = 0.002; partial η^2^ = 0.16), and a trend-level interaction between time and group (*F*_7,182_ = 2.1; *p* = 0.096; partial η^2^ = 0.075) in the absence of a main effect of group (*F*_1,26_ = 1.6; *p* = 0.21; partial η^2^ = 0.059). In contrast, there were no effects of time, group nor their interaction on amylase levels (Figure 3b; *F*s < 1.7; *p*s > 0.12; partial η^2^ values < 0.064).

BP (systolic and diastolic), pulse rate and skin conductance increased during both VR scenarios (Figure 3c–f), reflected in main effects of time for these measures (*F*s > 5.9; *p*s < 1 × 10^-6^; partial η^2^ values > 0.20) in the absence of main or interactive effects of group (*F*s < 1.3; *p*s > 0.19; partial η^2^ values < 0.064). For heart variability, there was a main effect of time (*F*_3,75_ = 3.6; *p* = 0.025; partial η^2^ = 0.13; Figure 3g) on the RMSSD measure, due to a decrease during the N-back epoch compared to the neighbouring time points (*p*s < 0.014), but no main or interactive effects of group (*F*s< 1.6; *p*s > 0.2; partial η^2^ values < 0.059), nor were there any group or time effects on the LF-HF measure (*F*s < 1.8; *p*s > 0.15; partial η^2^ values < 0.068; Figure 3h).

**Figure 4.**
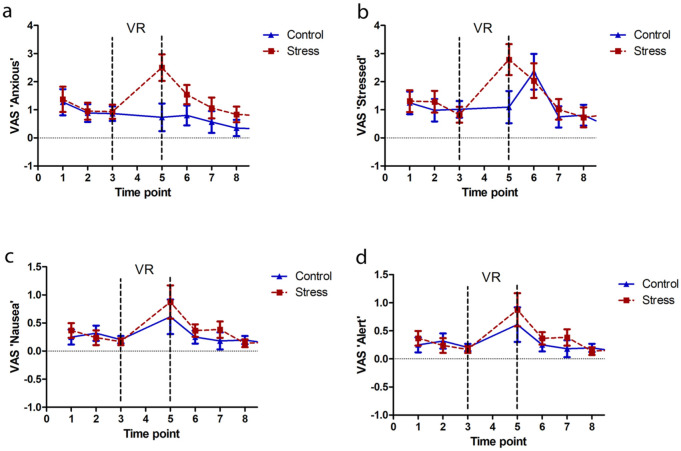
Variation in psychological indices across the experimental time course. Changes in VAS ratings of (a) ‘Anxious’, (b) ‘Stressed’, (c) ‘Nausea’ and (d) ‘Alert’ over the course of the experiment in those randomised to the control (blue triangles and solid lines) and stress (red squares and dashed lines) elevator. Note that VAS ratings were not taken at T4 as participants were in the VR. Vertical dashed lines indicate the period that participants were immersed in the VR environment (i.e. from just after time point 3 to just before time point 5).

Several of the psychological variables showed evidence of being affected by the VR environment, in a group-dependent manner in some cases. ‘Anxious’ ratings were selectively increased in the stress group following VR exposure, reflected in a time by group interaction (*F*_7,175_ = 2.5; *p* = 0.036; partial η^2^ = 0.090) that resulted from a group difference at T5 (post-VR; *p* = 0.005) but not at other time points (Figure 4a). There was also a main effect of time (*F*_7,175_ = 6.6; *p* = 6.4 × 10^-7^; partial η^2^ = 0.209) but no main effect of group (*F*_1,25_ = 0.88; *p* = 0.36; partial η^2^ = 0.034). There was a main effect of time on ‘Stressed’ ratings (Figure 4b; *F*_7,175_ = 10.8; *p* = 2.5 × 10^-11^; partial η^2^ = 0.303) in the absence of main or interactive effects of group (*F*s < 2.2; *p*s > 0.10; partial η^2^ values < 0.059). This resulted from higher ratings both after the VR and the N-back, compared to other time points (T5 differs from all other time points (*p*s < 0.005), except T6 (*p* = 0.62); T5 differs from all other others (*p*s < 0.026), except T6 and T1 (*p* = 0.061)). These data indicate that participants found both the VR (at least in the case of the stressful scenario, see above) and the N-back mildly stressful. Two other psychological ratings showed evidence of being slightly increased by VR exposure, namely ‘Nausea’ (Figure 4c) and ‘Alert’ (Figure 4d): there were main effects of time (*F*s > 3 .8; *p*s < 0.003; partial η^2^ values > 0.133) but no main or interactive effects of group (*F*s < 1; *p*s > 0.4; partial η^2^ values < 0.036) for either. For both ‘Nausea’ and ‘Alert’ ratings, there was an increase from T3 (final baseline) to T5 (post-VR; *p*s < 0.05). Finally, there was a main effect of time on VAS ‘Sad’ ratings (*F*_7,175_ = 2.6; *p* = 0.014; partial η^2^ = 0.096; data not shown), that appeared to result from a gradual decrease in ‘Sad’ ratings across the testing session (no individual time point comparisons reached significance), in the absence of main or interactive effects of group (*F*s < 1.2; *p*s > 0.35; partial η^2^ values < 0.044). There were no main or interactive effects on ratings of ‘Happy’ (*F*s < 1.2; *p*s > 0.3; partial η^2^ values < 0.044; data not shown).

We found very few correlations between stress indices of different types in our dataset (Supplementary Table 1). This applied both to a lack of correlations between psychological and physiological stress parameters, and also between one physiological measure and another.

## Discussion

Our data show that the VR stress scenario increased pulse rate, skin conductance, salivary cortisol and perceived stress and anxiety, and altered HRV but not BP, compared to the control. Alpha-amylase was numerically increased by exposure to the ‘stress’ scenario, compared with the control, but this did not reach formal statistical significance. Here, we comprehensively demonstrate physiological and psychological effects in individuals randomised to an active VR condition vs. a control VR condition. Thus, our findings underscore the utility of VR for inducing stress (and potentially other emotional states) for therapeutic and experimental purposes.

A number of previous studies have investigated the effect of VR environments on physiological stress measures but findings are mixed (reviewed in Diemer et al., 2014). Several have used height as a stressor but most of these focussed on comparing responses between groups (e.g. comparing healthy participants with a clinical population). Thus, whilst numerical increases in skin conductance and/or HR were observed over the course of VR height exposure in some of these studies (Cleworth et al., 2012; Diemer et al., 2016; Meehan et al., 2002; Meehan et al., 2005; Seinfeld et al., 2015; Wilhelm et al., 2005), these were not always examined statistically and it is possible that these changes resulted from exposure to the VR environment rather than stress per se. Only a few groups have counter-balanced the order of control vs. stressful height exposure within-subjects, and findings are similarly mixed within these. Thus, one, in which healthy participants undertook a beam walk in the VR environment at ground level vs. at height, found changes in HRV in the absence of effects on pulse or skin conductance (Peterson et al., 2018), whilst a second found changes in skin conductance but not pulse, when participants were asked to lean out over VR balconies of different heights (Simeonov et al., 2005). Our results clearly demonstrate increases in a number of physiological and psychological markers of stress during the VR height challenge using a randomised, between-subjects design. This approach, comparing active and control VR environments matched for movement demands, allows us to disambiguate the effects of stress vs. non-specific components arising from the VR environment per se, as well as to avoiding confounds due to cardiovascular demands and potential order effects.

A striking finding was that exposure to the stress VR paradigm was sufficient to induce changes in the HPA axis component of the stress response, viz. cortisol level, compared to the non-stress condition. VR versions of the Trier Social Stress Test, a widely used psychosocial stressor, increase salivary cortisol levels (e.g. Jönsson et al., 2010; Kelly et al., 2007; Kothgassner et al., 2016), consistent with the robust cortisol response elicited by the face-to-face version (Kirschbaum et al., 1993). However, few VR studies using non-socioevaluative stressors have investigated their effect on cortisol, and there is no clear evidence. Diemer and colleagues (Diemer et al., 2016) report that a subset of participants (20–30% of healthy controls, depending on the precise cut-off used) showed a cortisol response to a VR height challenge, but their results are difficult to interpret as they only report the proportion of responders and non-responders in their healthy control and acrophobia individuals. Therefore, to our knowledge, our study is the first to demonstrate an increase in cortisol levels following a non-socioevaluative VR stressor. We consider the finding somewhat surprising given the relatively brief nature of our stressor and the fact that our participants were all healthy controls. Unsurprisingly, the magnitude of the cortisol response was lower than that typically elicited by socioevaluative stressors (the stress condition elicited a maintenance of baseline cortisol levels, rather than the normal circadian decrease in this measure, which was seen in the control condition). However, our data provide further evidence for the utility of VR environments for inducing and modelling acute stress exposure in the laboratory setting.

A puzzling aspect of our findings is the directionality of our HRV results: specifically, RMSSD was increased, and the LF-HF ratio was decreased, in the stressor group, compared to the control group. This pattern is the opposite to what would be predicted following exposure to a stressor and, indeed, is opposite to what we have observed in a parallel study using a socioevaluative stressor (MM, PJH and EMT; unpublished observations). It is therefore unclear why we observe the pattern of responses shown here. However, as discussed briefly in the introduction, the relationship between HRV is complex and controversial (Billman, 2011; Billman, 2013; Shaffer and Ginsberg, 2017). For example, respiratory factors can influence HRV measures independent of changes in ANS activation (Billman, 2011). Thus, whilst our findings do not speak directly to this controversy, they do provide further evidence for a non-linear relationship between stress and HRV measures.

We found no group difference in N-back performance, in contrast to some previous studies that demonstrate effects of stress on memory performance (Henckens et al., 2009; Schoofs et al., 2008). However, findings are mixed: whilst the majority of studies suggest that stress impairs working memory (Duncko et al., 2009; Everaerd et al., 2006; Gärtner et al., 2014; Luethi et al., 2009; Schoofs et al., 2008), some find a beneficial effect (Cornelisse et al., 2011; Thompson and Morgan, 2013; Weerda et al., 2010) and others no differences ( al’Absi and Hoffman, 2004; Kuhlmann et al., 2005; Smeets et al., 2006). These variable findings likely reflect the complex relationship between stress and memory. Thus, as well as different types of memory being differentially affected by stress (Cornelisse et al., 2011; Kuhlmann et al., 2005; Schwabe et al., 2012), there are important effects of, for example, gender (Cornelisse et al., 2011; Zandara et al., 2016) and the timing of the cognitive task relative to the stressor (Schwabe et al., 2012). It should be noted that our VR stress paradigm did not involve a socioevaluative component and was relatively mild in nature, inducing only a relatively brief cardiovascular and cortisol response compared to, for example, the Trier Social Stress Test (Allen et al., 2014), and so may have been insufficient to induce stress-related changes in working memory. However, whilst some previous studies have shown associations between stress-related cortisol changes and working memory performance (Schoofs et al., 2008), others have observed stress-related changes in working memory in the absence of group differences in cortisol levels (Duncko et al., 2009). Thus, although our findings suggest that mild stress does not affect working memory performance in young, healthy men, further studies are required to clarify the relationships between stressor, stress response, and working memory.

At first sight, the absence of correlations between different measures of the stress response may seem surprising. However, there is a substantial literature arguing against the ‘stress response’ as being a unitary construct, and weak or absent correlations between physiological and psychological measures of the stress response is a common finding both in experimental and naturalistic settings (Campbell and Ehlert, 2012; Dickerson and Kemeny, 2004; Mauss et al., 2005). Our findings emphasise the value of examining multiple variables to establish a full picture, not least since different components of the stress response may impact differentially on outcomes of interest.

Our study has some limitations. Firstly, we investigated a relatively small group of healthy young adult male volunteers. Thus, it is not clear to what extent our findings can be generalised to females, other ages, or to patient groups (Cornelisse et al., 2011; Diemer et al., 2016). Secondly, our stressor was brief and relatively mild; therefore, the psychological and physiological effects we observe are relatively modest in terms of magnitude and duration. Accordingly, it is not clear that our findings will be of relevance to situations of more extreme or prolonged stress. Broadly speaking, any attempt to extrapolate our findings should be mindful of the complex interplay between psychological and physiological effects of stress, its context, and the cognitive impacts (Schwabe et al., 2012).

In conclusion, we have demonstrated that a VR environment can be used to induce a physiologically and psychologically relevant stress response. Therefore, our findings underscore the utility of scenarios of this type for therapeutic purposes, for example to allow individuals to experience an aversive environment in a controlled, safe setting as an adjunct to or alternative means of delivering exposure therapy (Freeman et al., 2019). The fact that we observed a measurable stress response even in healthy individuals points to the potential of VR scenarios in an experimental medicine setting, both as a model in which to test putative anxiolytics and as a tool to investigate how stress might affect the therapeutic actions of drugs of interest (Diemer et al., 2013; Donahue et al., 2009). As VR scenarios become increasingly realistic and diverse, and the technology ever more cost-effective, its use in research and clinical settings will likely increase accordingly. Our findings suggest that VR will be of utility in both situations.

## Supplemental Material

Supplementary_table – Supplemental material for It feels real: physiological responses to a stressful virtual reality environment and its impact on working memory Supplemental material, Supplementary_table for It feels real: physiological responses to a stressful virtual reality environment and its impact on working memory by Marieke AG Martens, Angus Antley, Daniel Freeman, Mel Slater, Paul J Harrison and Elizabeth M Tunbridge in Journal of Psychopharmacology
