# Supplementary material for: It feels real: physiological responses to a stressful virtual reality environment and its impact on working memory
Source: J Psychopharmacol. 2019 Jul 11;33(10):1264–73. doi: 10.1177/0269881119860156 (PMC6764008; doi:10.1177/0269881119860156)
Supplement: Supplementary_table – Supplemental material for It feels real: physiological responses to a stressful virtual reality environment and its impact on working memory [file Supplementary_table.pdf]

		Locus of control	Baseline VAS 'Stressed'	Baseline VAS 'Anxious'	Post-VR VAS 'Stressed'	Post-VR VAS 'Anxious'	Mid-VR verbal stress rating	Cortisol (% baseline)	Alpha-amylase (% baseline)	Systolic BP (% baseline)	Diastolic BP (% baseline)	Pulse (% baseline)	Skin conductance (% baseline)	HRV RMSSD (% baseline)	HRV LF-HF ratio (% baseline)	0 back % correct	1 back % correct	2 back % correct	3 back % correct
Self esteem	Spearman's rho	.688**	-0.189	-0.262	-0.157	-0.187	-0.026	-0.131	0.138	-0.043	-0.229	-0.045	0.100	0.192	-0.359	0.132	.387*	0.061	-0.112
	p value	0.000	0.335	0.179	0.436	0.350	0.896	0.506	0.483	0.833	0.250	0.824	0.636	0.338	0.066	0.510	0.046	0.764	0.579
Locus of control	Spearman's rho	1.000	-0.312	-0.328	-0.273	-0.260	-0.086	-0.069	-0.201	-0.201	-0.044	-0.118	0.112	0.178	-0.090	0.227	.433*	0.217	0.053
	p value		0.106	0.089	0.169	0.190	0.663	0.728	0.305	0.316	0.827	0.556	0.594	0.373	0.656	0.255	0.024	0.277	0.793
Baseline VAS 'Stressed'	Spearman's rho		1.000	.761**	.665**	.612**	0.138	-0.371	-0.189	0.073	-0.093	0.151	0.067	0.239	-0.183	0.024	-0.159	-0.033	-0.262
	p value			0.000	0.000	0.001	0.483	0.052	0.336	0.716	0.644	0.451	0.751	0.230	0.361	0.907	0.428	0.869	0.187
Baseline VAS 'Anxious'	Spearman's rho			1.000	.533**	.547**	0.051	-.397*	-0.027	0.039	0.079	-0.046	-0.057	0.155	-0.085	-0.115	-0.181	-0.004	-0.104
	p value				0.004	0.003	0.797	0.036	0.892	0.846	0.695	0.820	0.786	0.441	0.674	0.568	0.366	0.982	0.606
Post-VR VAS 'Stressed'	Spearman's rho				1.000	.926**	.591**	-0.058	0.051	0.130	-0.279	0.214	0.167	0.328	-0.233	0.102	-0.196	0.033	-0.077
	p value					0.000	0.001	0.773	0.801	0.527	0.167	0.295	0.436	0.102	0.252	0.621	0.336	0.872	0.710
Post-VR VAS 'Anxious'	Spearman's rho					1.000	.587**	0.079	0.004	0.072	-0.295	0.127	0.262	.428*	-0.344	0.051	-0.111	0.101	0.026
	p value						0.001	0.695	0.986	0.726	0.143	0.535	0.216	0.029	0.085	0.804	0.589	0.624	0.899
Mid-VR verbal stress rating	Spearman's rho						1.000	0.312	0.359	0.159	0.067	.395*	.415*	.490**	-0.360	0.269	0.108	0.223	0.157
	p value							0.106	0.061	0.427	0.740	0.041	0.039	0.010	0.065	0.174	0.594	0.263	0.435
Cortisol (% baseline)	Spearman's rho							1.000	0.165	0.108	0.167	.439*	0.393	0.180	-0.114	0.047	-0.032	-0.114	-0.002
	p value								0.402	0.591	0.406	0.022	0.052	0.369	0.571	0.815	0.873	0.572	0.990
Alpha-amylase (% baseline)	Spearman's rho								1.000	0.262	0.198	0.106	0.156	-0.081	-0.190	0.050	.392*	0.194	0.159
	p value									0.187	0.323	0.598	0.456	0.687	0.341	0.804	0.043	0.333	0.429
Systolic BP (% baseline)	Spearman's rho									1.000	0.329	0.220	0.341	0.175	-0.168	0.011	0.081	0.192	0.169
	p value										0.094	0.271	0.103	0.382	0.403	0.959	0.695	0.347	0.408
Diastolic BP (% baseline)	Spearman's rho										1.000	0.294	0.100	0.089	0.049	0.006	0.171	0.146	0.273
	p value											0.136	0.642	0.661	0.809	0.978	0.404	0.476	0.177
Pulse (% baseline)	Spearman's rho											1.000	0.362	0.173	-0.130	-0.080	-0.230	-0.269	-0.274
	p value												0.082	0.389	0.518	0.697	0.259	0.184	0.176

Skin conductance (% baseline)	Spearman's rho												1.000	.447*	-0.264	0.102	0.312	0.042	0.306
	p value													0.029	0.212	0.629	0.129	0.841	0.137
HRV RMSSD (% baseline)	Spearman's rho													1.000	-.700**	0.308	-0.009	0.152	0.145
	p value														0.000	0.126	0.967	0.459	0.478
HRV LF-HF ratio (% baseline)	Spearman's rho														1.000	-0.090	-0.120	-0.074	-0.089
	p value															0.663	0.558	0.721	0.664
0 back % correct	Spearman's rho															1.000	.497**	.444*	.423*
	p value																0.008	0.020	0.028
1 back % correct	Spearman's rho																1.000	.623**	.510**
	p value																	0.001	0.007
2 back % correct	Spearman's rho																	1.000	.583**
	p value																		0.001
3 back % correct	Spearman's rho																		1.000
	p value																		
